# Ultrasound characteristics comparison and development of a predictive nomogram for intraductal papilloma and ductal carcinoma *in situ*: a retrospective cohort study

**DOI:** 10.3389/fonc.2025.1454951

**Published:** 2025-04-17

**Authors:** Liyang Su, Qiaojie Xie, Aling Yi, Qingquan Zhang, Jinzhen Chen

**Affiliations:** Department of Ultrasonography, Quanzhou First Hospital Affiliated to Fujian Medical University, Quanzhou, China

**Keywords:** intraductal papilloma, ductal carcinoma *in situ*, ultrasound, artificial intelligence, breast cancer

## Abstract

**Background:**

Intraductal Papilloma (IDP) and Ductal Carcinoma *In Situ* (DCIS) are significant benign and pre-invasive breast lesions, respectively. This study aimed to investigate ultrasound features and develop a predictive nomogram for discriminating between IDP and DCIS.

**Methods:**

Conducted at Quanzhou First Hospital over a three-year period, 389 patients were enrolled with detailed ultrasound examinations and confirmed pathological diagnoses. IDP was classified into Grades 3, 4, and 5, whereas DCIS presented with a mass-like morphology. Patients meeting the inclusion criteria underwent rigorous analysis, with exclusion criteria eliminating those with incomplete imaging data or confounding comorbidities. Ultrasound characteristics, including lesion size, shape, margin, and echogenicity, etc., were systematically evaluated and compared between the two groups. Univariate and multivariate logistic regression analyses were conducted to identify significant risk factors. Subsequently, based on these characteristics, both static and dynamic nomograms were developed. The performance of the nomograms was evaluated using the area under the receiver operating characteristic curve (AUC), calibration plots, and decision curve analysis (DCA).

**Results:**

The study cohort included 272 patients in the training set and 117 in the validation set. Significant differences were observed between IDP and DCIS in age, size, shape, aspect ratio, margin, duct dilatation, and microcalcification (*P* < 0.05). Logistic regression analyses identified age, size, aspect ratio, margin, microcalcification, and duct dilatation as independent risk factors. Compared to DCIS, IDP is associated with younger age, smaller size, clearer margins, fewer microcalcifications, and more ductal dilation. The performance of the nomogram developed to predict IDP and DCIS showed an AUC of 0.918 in the training set and 0.888 in the validation set. The calibration curve indicates a strong fit of the predictive model in the validation set, with the Hosmer-Lemeshow test showing high consistency between predicted and actual probabilities (training set, *P* = 0.875; validation set, *P* = 0.751). Additionally, DCA confirms the clinical utility of the model.

**Conclusion:**

The nomogram incorporating key predictors provides a valuable tool for differentiating between IDP and DCIS based on ultrasound characteristics. This approach aids in clinical decision-making and potentially reduces unnecessary biopsies.

## Introduction

Breast cancer has emerged as the most diagnosed type of cancer globally in 2020, with an addition of 2.3 million new cases (1). Prior to the implementation of screening, ductal carcinoma *in situ* (DCIS) was rarely identified. Presently, DCIS constitutes approximately 20-33% of all detected breast cancer cases (2–4). DCIS is characterized by tumor-like cell proliferation within the ductal lobular structures of the breast, without invading the myoepithelial basement membrane (5). It can be classified as low, intermediate, or high grade, with higher grades being more prone to progression to invasive breast cancer (IBC) (6). While DCIS itself is not life-threatening and can remain asymptomatic, the proportions that progress to IBC and those that regress if left untreated are still unclear. A 2019 U.S. retrospective study found that 10-15% of women with untreated DCIS developed invasive cancer after a median follow-up of 5.5 years (7). Ultrasonographic images of DCIS exhibit a wide spectrum from a mass to a non-mass presentation (8, 9). Image-based classification of 705 cases of DCIS showed that non-mass abnormalities accounted for 60% of all lesions, while masses constituted 40%. Although there is a current trend highlighting the issues of overdiagnosis and overtreatment of DCIS, choosing not to treat it is considered unethical (10).

Intraductal papilloma (IDP) represents a high-risk benign breast condition localized within the ductal system, demonstrating a propensity for potential carcinogenic evolution. IDP accounts for approximately 10% of all benign growths within the breast (11). While the incidence rate of IDP among women is estimated to range from 2 - 3%, the risk of developing IDP increases to 40-70% in women who present with nipple discharge (12). Ultrasonography functions as the principal diagnostic modality enabling the classification of IDP into five distinct ultrasound grades based on its occurrence and development within the ducts (13). Grade 1 necessitates close observation, categorized as BI-RADS 3. Grades 2, 3, and 4 recommend selective surgery, classified as BI-RADS 4A. Grade 5 calls for immediate surgical intervention, classified as BI-RADS 4B.

The diagnosis of mass-forming DCIS poses challenges in ultrasound imaging, particularly with Grade 3, 4, and 5 IDP. Current diagnostic tools, including mammography and ultrasound, often struggle to accurately differentiate between these two conditions, which can lead to either overdiagnosis or underdiagnosis. To my knowledge, there have been no detailed studies specifically addressing the ultrasound or mammographic characteristics of these two tumors. A discussion of these diagnostic challenges underscores the importance of developing more accurate predictive models to assist in clinical decision-making.

Recent studies have developed nomograms and predictive models have been developed for breast lesions, including the Gail Model, Tyrer-Cuzick Model, and various machine learning approaches. However, these existing models often focus on general risk assessment rather than specifically distinguishing between IDP and low-grade DCIS, which is critical for accurate diagnosis and treatment planning (14). The predictive models of nomograms, which incorporate various factors such as ultrasound characteristics, patient demographics, and potentially other imaging modalities, show significant promise for enhancing diagnostic accuracy (15). Nomograms offer a visual representation of predictive models, serving as a practical tool for clinicians to estimate the likelihood of a specific clinical event or diagnosis. This study aims to compare the ultrasound features of IDP and DCIS and establish a predictive nomogram to enhance diagnostic accuracy.

## Materials and methods

### Participants and study design

The study was conducted at Quanzhou First Hospital over a three-year period (January 2021 to December 2023). A total of 389 patients were rigorously enrolled, consisting of 210 cases of IDP and 179 cases of DCIS. Each patient underwent a detailed ultrasound examination followed by a confirmed pathological diagnosis through biopsy or surgical pathology. In our study, biopsy data were confirmed through histopathological examination conducted by experienced pathologists.

Inclusion criteria comprised patients diagnosed with IDP or DCIS who had undergone both ultrasound imaging and definitive pathological assessment, with complete medical records for analysis. IDP was categorized as Grades 3, 4, and 5, whereas DCIS presented as a mass-like morphology. The ultrasound grading criteria for IDP are as follows (13): Grade 1: Characterized by ductal dilation devoid of echogenic protuberances. Grade 2: Shows ductal dilation with echogenic protuberances. Grade 3: Exhibits the presence of a complex mass within the breast gland with internal echogenic protuberances. Grade 4: Involves the identification of a hypoechoic mass within the breast gland. Grade 5: Indicates visualization of a hypoechoic mass within the breast gland along with rich color Doppler signals, signaling a likelihood of developing microinvasive intraductal papillary carcinoma.

**Figure 1:**
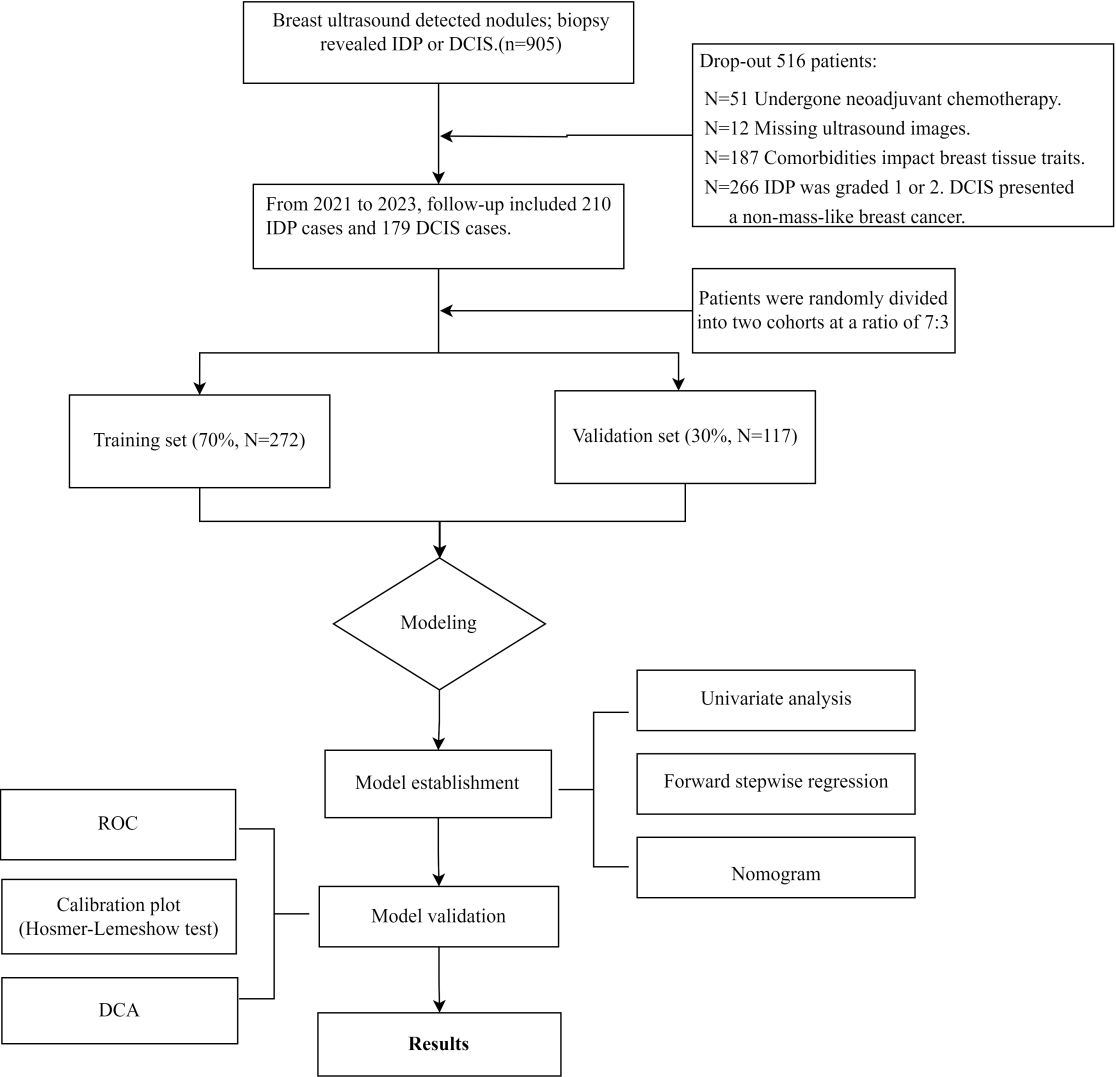
Flowchart of this study.

Exclusion criteria included patients with incomplete ultrasound imaging data, those with a history of prior breast surgery that could potentially affect current imaging interpretations, individuals with comorbidities influencing breast tissue characteristics, and patients presenting with severe complications. Additionally, patients with breast cancer recurrence or those undergoing chemotherapy for malignant breast tumors were excluded from the study. Exclude all cases of breast cancer that present as microcalcifications, diffuse changes, or other characteristics that do not conform to mass-like morphology on imaging studies (16) (**Figure 1**).

This study received approval from the ethical review board of Quanzhou First Hospital (Approval No. [2024] K129). All experimental procedures were conducted in strict adherence to the principles outlined in the Declaration of Helsinki and other relevant guidelines. Since this study was a retrospective analysis, the requirement for informed consent was waived by the ethical review board.

### Instruments and methods

The PHILIPS (Affiniti50, EPIQ 7) color ultrasonic diagnostic apparatus with linear array probe (4–12 MHz frequency) was used.

To ensure a comprehensive evaluation, patients were positioned supine, enabling optimal exposure of breasts for a detailed ultrasound examination. The acquired ultrasound images underwent systematic assessment following the well-defined standards set forth by the Breast Imaging-Reporting and Data System (BI-RADS). Special attention was devoted to retaining slices displaying notable nodular ultrasound characteristics, capturing details such as size, shape, margin clarity, echogenicity in relation to surrounding tissues, internal echoes, aspect ratio, posterior features, calcifications, echo edge, duct dilatation, Color Doppler Flow Imaging (CDFI) grading to evaluate vascularity, and nodular resistance index (RI) with meticulous precision.

### Ultrasound evaluation

A retrospective analysis of all images was conducted by two ultrasound physicians with 10 years of experience in breast ultrasound diagnosis, respectively. They were blinded to the pathological results of the nodules to ensure unbiased evaluation. CDFI was utilized for semi-quantitative assessment, using the Adler method (17) to categorize the nodular blood flow signals into four levels: Level 0 indicating absence of blood flow within the nodules, Level 1 denoting minimal blood flow with 1-2 rod-shaped or punctate vessels within the nodules, Level 2 representing moderate blood flow with 1 long or 3-4 punctate vessels, and Level 3 indicating abundant blood flow with 2 long vessels or 5 or more punctate vessels within the nodules. If discrepancies arise between the two ultrasound physicians’ evaluations, a senior physician with extensive experience will be consulted to make the final determination. Additionally, the kappa coefficient will be calculated and presented in the results section to quantitatively assess the agreement between the physicians.

### Statistical analysis

Statistical analyses were conducted using R software (version 4.3.2). Quantitative data were analyzed with t-tests for normal distributions and the Mann-Whitney U Test for non-normal distributions. Categorical data were compared using chi-square or Fisher’s Exact Test. Logistic regression identified ultrasound features associated with IDP or DCIS. A predictive nomogram was constructed using selected features, and its performance was evaluated using ROC curves, with diagnostic metrics including sensitivity, specificity, positive predictive value (PPV), and negative predictive value (NPV). Calibration and clinical benefits were assessed, and model fit was evaluated with the Hosmer-Lemeshow test. Inter-observer reliability was assessed using the Kappa statistic, and a dynamic nomogram was created online. Significance was set at *P* < 0.05.

## Results

### Basic characteristics

The overall study cohort comprised a total of 389 patients, with 272 patients designated to the training set and 117 patients to the validation set. The inter-rater reliability between the two ultrasound physicians was calculated using the kappa statistic. A kappa value of 0.86 was obtained, indicating a substantial agreement between the observers. In the training set, there were no significant statistical differences (*P* > 0.05) between the two groups in terms of height, weight, clinical symptoms, palpation, posterior echo, hyperechoic halo, internal flow, and RI. However, we identified statistically significant differences (*P* < 0.05) in age, size, shape, aspect ratio, margin, architectural distortion, and microcalcification. Similarly, in the validation set, we observed no significant differences (*P* > 0.05) for height, weight, clinical symptoms, palpation, posterior echo, aspect ratio, architectural distortion, hyperechoic halo, internal flow, peripheral flow, and RI, while age, size, shape, margin, microcalcification, posterior echo, and duct dilatation showed significant statistical differences (*P* < 0.05). The characteristics and outcomes of both IDP and DCIS within the training and validation sets have been systematically enumerated and are presented in detail in **Table 1**.

**Table 1:** Baseline characteristics in the training and validation sets.

	Training set		*P*	Validation set		*P*
	IDP	DCIS		IDP	DCIS	
	N=147	N=125		N=63	N=54	
Age (years)	45.0 ± 8.22	47.7 ± 9.54	0.014	44.4 ± 10.3	49.3 ± 8.47	0.005
Height (cm)	159 ± 5.35	158 ± 4.77	0.218	159 ± 4.72	157 ± 5.03	0.177
Weight (kg)	59.8 ± 11.4	62.5 ± 45.5	0.518	57.8 ± 7.90	57.5 ± 8.83	0.814
Clinical symptoms			0.525			0.252
Symptomatic	101 (75.9%)	100 (80.0%)		43 (76.8%)	47 (87.0%)	
Asymptomatic	32 (24.1%)	25 (20.0%)		13 (23.2%)	7 (13.0%)	
Palpation			0.159			1
Palpable	40 (30.1%)	27 (21.6%)		12 (21.4%)	11 (20.4%)	
Unpalpable	93 (69.9%)	98 (78.4%)		44 (78.6%)	43 (79.6%)	
Size(cm)	1.16 (0.60)	2.51 (1.77)	<0.001	1.49 (1.05)	2.32 (1.77)	0.003
Shape			<0.001			<0.001
Regular	84 (57.1%)	16 (12.8%)		36 (57.1%)	9 (16.7%)	
Irregular	63 (42.9%)	109 (87.2%)		27 (42.9%)	45 (83.3%)	
Aspect ratio			<0.001			0.14
<1	124 (84.4%)	123 (98.4%)		53 (84.1%)	51 (94.4%)	
≥1	23 (15.6%)	2 (1.60%)		10 (15.9%)	3 (5.56%)	
Margin			<0.001			<0.001
Distinct	97 (66.0%)	24 (19.2%)		49 (77.8%)	13 (24.1%)	
Indistinct	50 (34.0%)	101 (80.8%)		14 (22.2%)	41 (75.9%)	
Architectural distortion			0.041			0.175
No	139 (94.6%)	124 (99.2%)		56 (88.9%)	52 (96.3%)	
Yes	8 (5.44%)	1 (0.80%)		7 (11.1%)	2 (3.70%)	
Microcalcification			<0.001			<0.001
No	124 (84.4%)	49 (39.2%)		51 (81.0%)	22 (40.7%)	
Yes	23 (15.6%)	76 (60.8%)		12 (19.0%)	32 (59.3%)	
Posterior echo			0.607			0.685
Other echoes	138 (93.9%)	120 (96.0%)		59 (93.7%)	52 (96.3%)	
Attenuation	9 (6.12%)	5 (4.00%)		4 (6.35%)	2 (3.70%)	
Hyperechoic halo			0.127			1
No	143 (97.3%)	125 (100%)		60 (95.2%)	51 (94.4%)	
Yes	4 (2.72%)	0 (0.00%)		3 (4.76%)	3 (5.56%)	
Duct dilatation			<0.001			0.014
No	75 (51.0%)	108(86.4%)		34 (54.0%)	27 (81.8%)	
Yes	72 (49.0%)	17 (13.6%)		29 (46.0%)	6 (18.2%)	
Internal flow			1			0.179
Alder 0-I	134 (91.8%)	114 (91.2%)		49 (77.8%)	48 (88.9%)	
Alder II-III	12 (8.22%)	11 (8.80%)		14 (22.2%)	6 (11.1%)	
Peripheral flow			0.023			0.184
Alder 0-I	138 (94.5%)	107 (85.6%)		60 (95.2%)	47 (87.0%)	
Alder II-III	8 (5.48%)	18 (14.4%)		3 (4.76%)	7 (13.0%)	
RI	0.70 (0.17)	0.74 (0.11)	0.529	0.79 (0.30)	0.73 (0.18)	0.826

### Logistic regression analysis in the training set and construction of nomogram

In the univariate logistic regression analyses, seven candidate variables, age, size, shape, aspect ratio, margin, microcalcification, and duct dilatation, were significantly associated with both IDP and DCIS (*P* < 0.05). Further exploration through multivariate forward stepwise logistic regression identified age (*P* = 0.006), size (*P* < 0.001), aspect ratio (*P* = 0.016), margin (*P* = 0.016), microcalcification (*P* = 0.004), and duct dilatation (*P* < 0.001) as independent risk factors (**Table 2**). Compared to DCIS, IDP was associated with younger age, smaller size, clearer margins, fewer microcalcifications, and more ductal dilation.

**Table 2:** Univariate and multivariate logistic regression analysis in the training set.

Characteristics	Univariate analysis				Multivariate analysis			
	B	OR	95%CI	*P*	B	OR	95%CI	*P*
Age	0.035	1.04	1.01-1.06	0.013	0.055	1.06	1.02-1.1	0.006
Height	-0.013	0.99	0.94-1.03	0.583	NA	NA	NA	NA
Weight	0.003	1	0.99-1.01	0.507	NA	NA	NA	NA
Clinical symptoms	-0.232	0.79	0.44-1.42	0.433	NA	NA	NA	NA
Palpation	0.513	1.67	0.96-2.9	0.068	NA	NA	NA	NA
Size	1.125	3.08	2.2-4.33	<0.001	0.935	2.55	1.62-4	<0.001
Shape	2.222	9.23	4.97-17.15	<0.001	0.852	2.34	0.99-5.58	0.054
Aspect ratio	-2.442	0.09	0.02-0.38	0.001	-1.996	0.14	0.03-0.69	0.016
Margin	2.089	8.08	4.61-14.15	<0.001	1.024	2.79	1.21-6.39	0.016
Architectural distortion	-1.972	0.14	0.02-1.13	0.065	NA	NA	NA	NA
Microcalcification	2.168	8.74	4.9-15.59	<0.001	1.184	3.27	1.47-7.24	0.004
Posterior echo	-0.455	0.63	0.21-1.95	0.426	NA	NA	NA	NA
Hyperechoic halo	-15.439	0	0-Inf	0.983	NA	NA	NA	NA
Duct dilatation	-1.822	0.16	0.09-0.3	<0.001	-1.726	0.18	0.07-0.43	<0.001
Internal flow	0.075	1.08	0.46-2.53	0.864	NA	NA	NA	NA
Peripheral flow	1.065	2.9	1.22-6.93	0.056	NA	NA	NA	NA

NA, Not Applicable.

**Figure 2:**
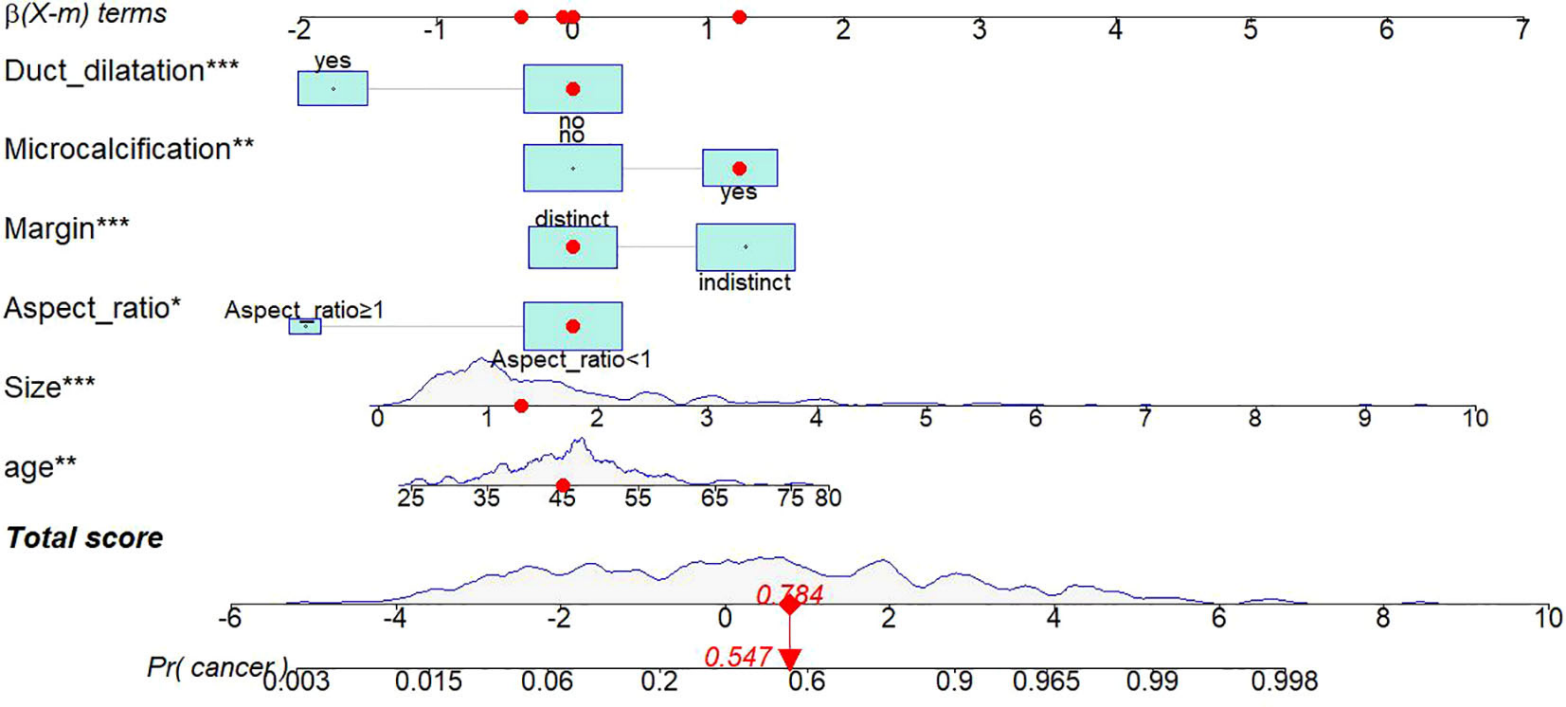
Static nomogram model. The nomogram was developed based on logistic regression analysis results to predict the diagnosis of IDP from DCIS. The dynamic nomogram can be accessed at https://suliyang.shinyapps.io/IDP-DCIS/. This model comprises age, lesion size, aspect ratio, margin characteristics, microcalcifications, and duct dilatation. *p < 0.05, **p < 0.01, ***p < 0.001.

Thereafter, a static nomogram and an online dynamic online nomogram (https://suliyang.shinyapps.io/IDP-DCIS/) were developed by incorporating these seven predictors(**Figure 2**).

### Evaluation of nomogram

**Figure 3:**
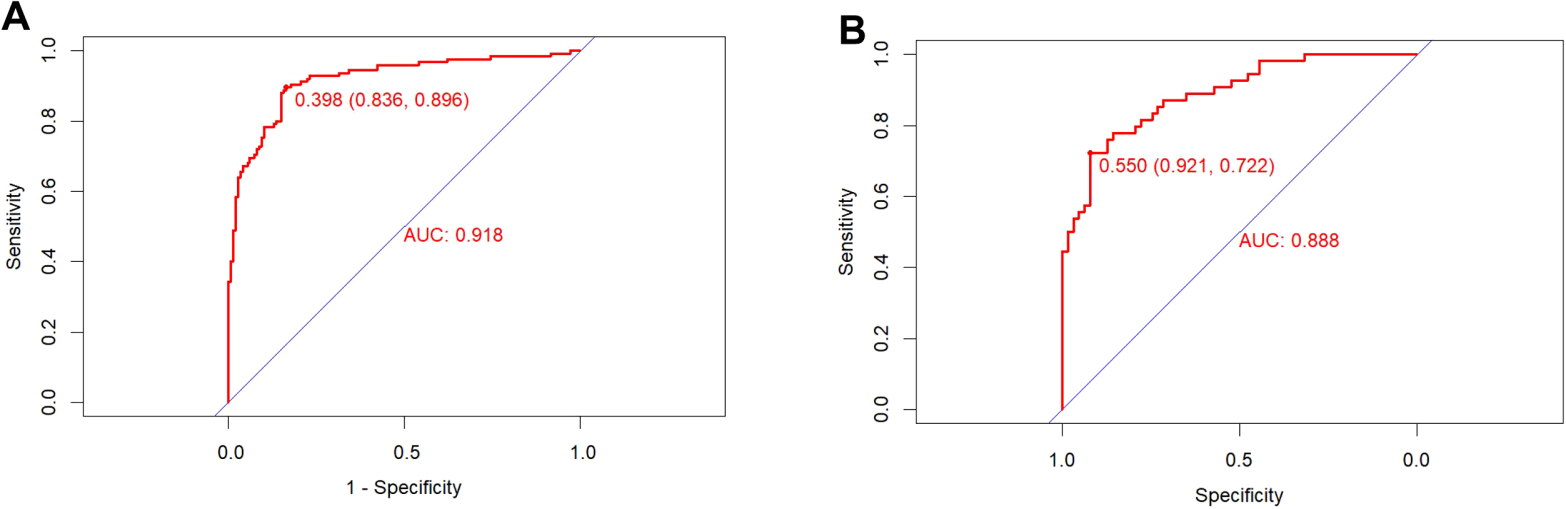
ROC curve of the training set **(A)** and validation set **(B)**. The pooled area under the ROC of the nomogram for the predictive model is 0.918 in the training set and 0.888 in the validation set, indicating moderately good performance.

Using the ROC curve to assess the discriminative ability of a predictive model. The area under the ROC curve of the predictive model is 0.918 in the training set and 0.888 in the validation set, indicating excellent performance (**Figure 3**). The predictive model is calibrated using calibration plots and the Hosmer-Lemeshow test. In the validation set, the model demonstrated a sensitivity of 86%, a specificity of 82%, a PPV of 87%, and a NPV of 83%.

**Figure 4:**
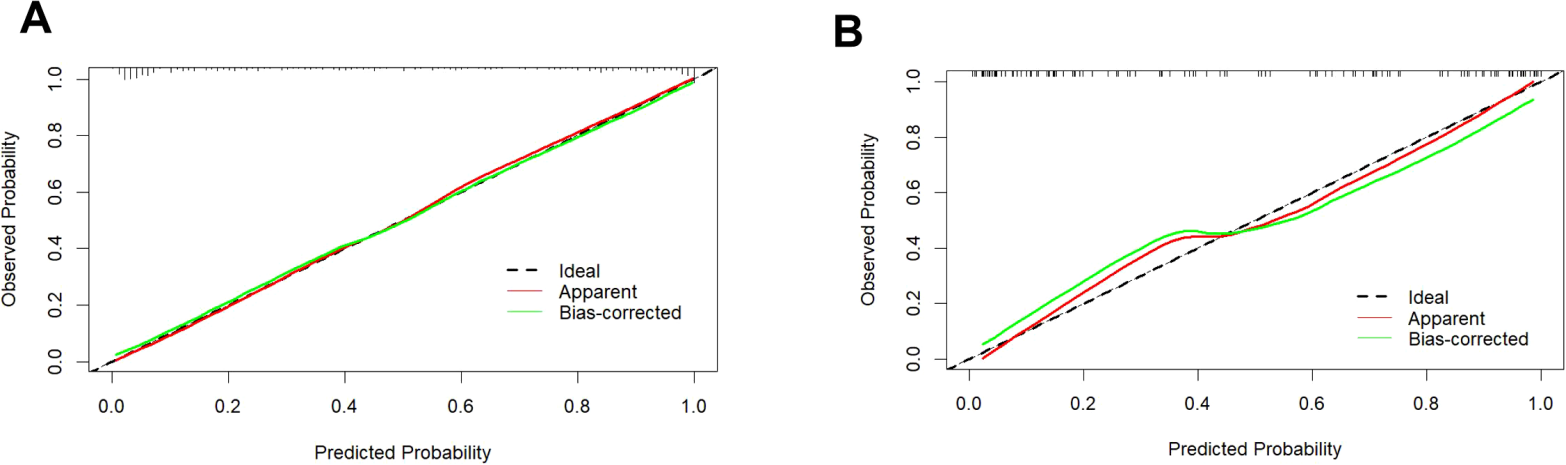
Calibration curve of the training set **(A)** and validation set **(B)**. Calibration curves depict the correlation between the predicted probability (x-axis) and the actual probability (y-axis). The red line along the diagonal signifies where predicted probability equals actual probability, while the green line represents the calibration curve of the nomogram. Both the training and validation set curves closely align with the dashed line, demonstrating high calibration accuracy.

**Figure 5:**
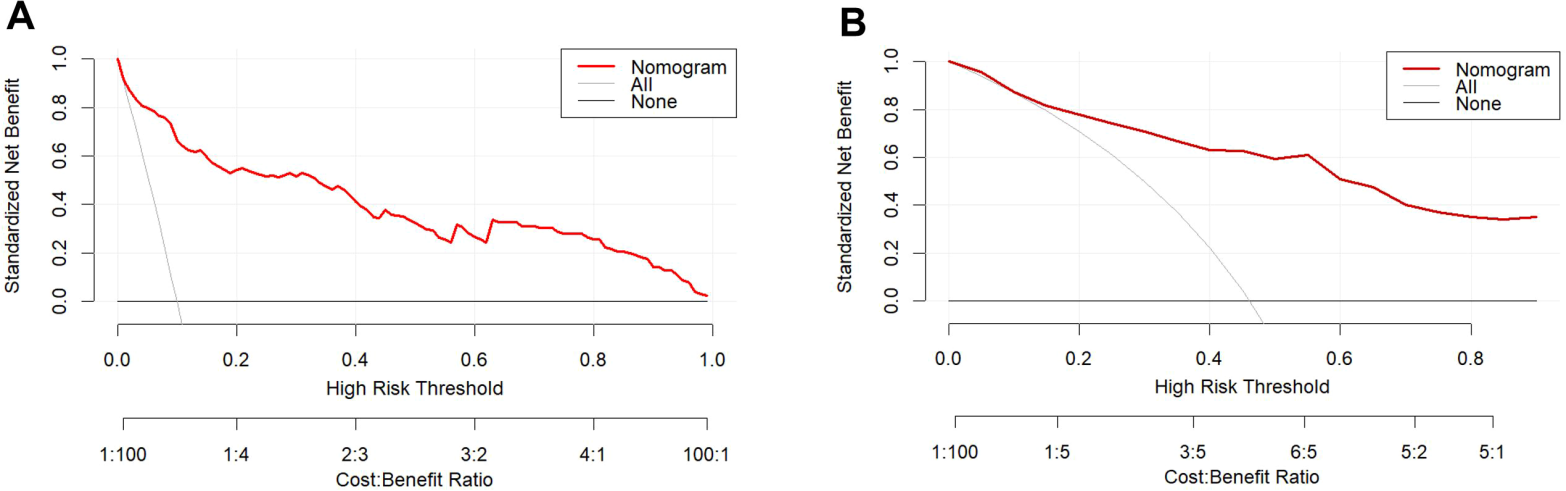
DCA of the training set **(A)** and validation set **(B)**. The x-axis displays the threshold probability, while the y-axis quantifies the net benefit. The DCA curve values lie above the lines for None and All, indicating acceptable model performance within this range.

**Figure 6:**
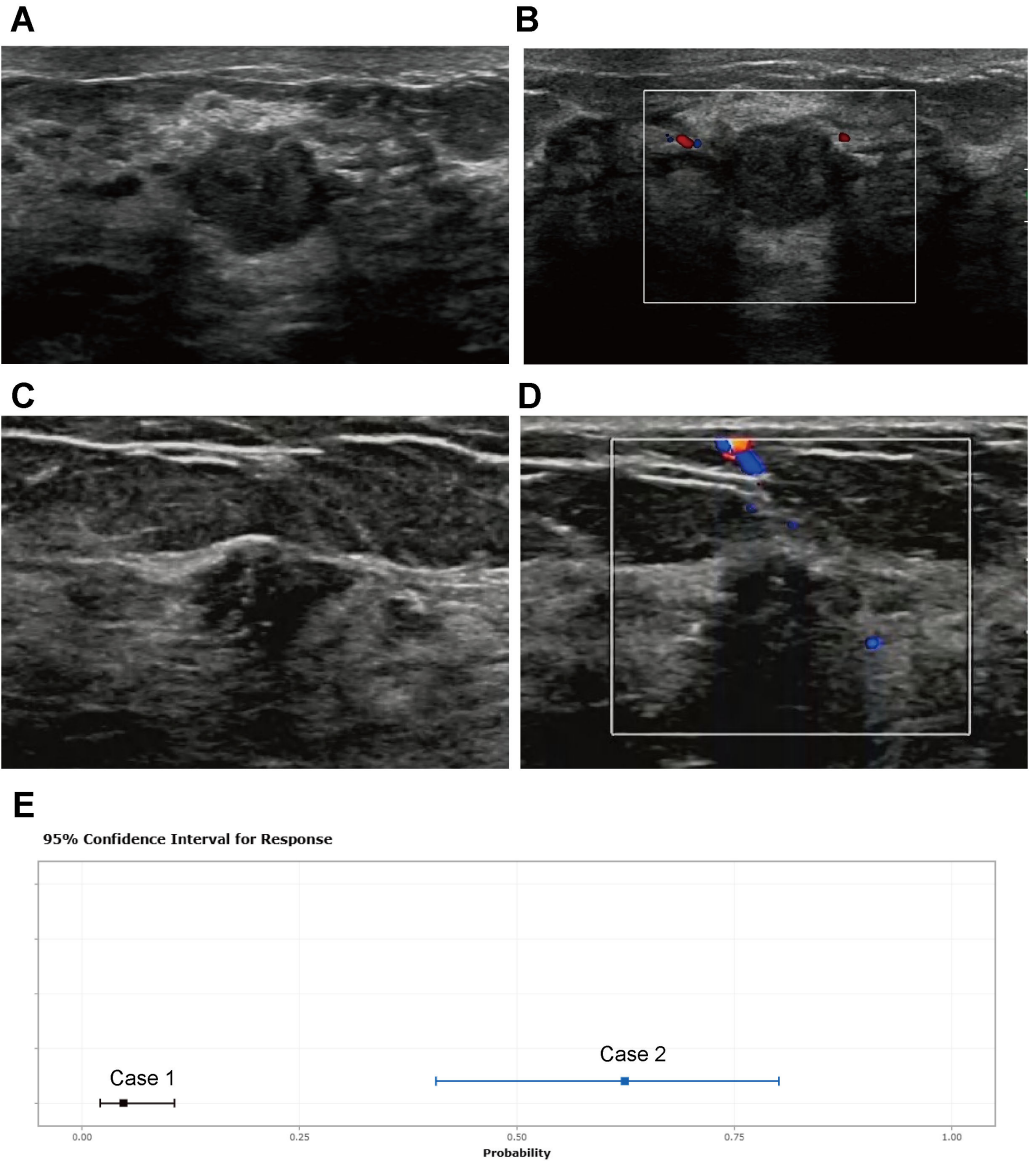
Two cases whose outcomes were successfully predicted by the nomogram model. Case 1 **(A, B)** A 46-year-old patient presented with ultrasound characteristics of distinct margins, aspect ratio < 1, absence of microcalcifications, and ductal dilation, with a predicted probability of 4.78% (95% CI: 2.07%-10.63%). Case 2 **(C, D)** A 55-year-old patient exhibited ultrasound findings of indistinct margins, aspect ratio > 1, presence of microcalcifications, and no ductal dilation, with a predicted probability of 62.4% (95% CI: 40.7%-80.1%). **(E)** shows the calculated probabilities from the dynamic nomogram for the above two cases.

The calibration curve illustrates a strong fit of the predictive model in the validation set. Further validation through the Hosmer-Lemeshow test demonstrates high consistency between predicted and actual probabilities (training set, *P* = 0.875; validation set, *P* = 0.751) (**Figure 4**). DCA confirms the clinical utility of the model (**Figure 5**). Two cases were successfully predicted by the nomogram model (**Figure 6**). Case 1 (**Figures 6A, B**) involved a 46-year-old patient who exhibited ultrasound characteristics of distinct margins, an aspect ratio of less than 1, absence of microcalcifications, and ductal dilation, with a predicted probability of 4.78% (95% CI: 2.07%-10.63%). Case 2 (**Figures 6C, D**) featured a 55-year-old patient with ultrasound findings of indistinct margins, an aspect ratio greater than 1, the presence of microcalcifications, and no ductal dilation, with a predicted probability of 62.4% (95% CI: 40.7%-80.1%). **Figure 6E** shows the calculated probabilities from the dynamic nomogram for the two cases described above.

## Discussion

The timing and approach to surgery for breast DCIS and IDP differ (18), thus, accurate identification of breast DCIS is crucial during screening and preoperative diagnosis processes. Our analyses revealed several critical ultrasound features that significantly differentiate IDP from DCIS and serve as independent predictors for the presence of breast cancer. In the univariate logistic regression analyses, our findings identified age, lesion size, shape, aspect ratio, margin characteristics, presence of microcalcifications, and duct dilatation as significant variables associated with both IDP and DCIS. These variables demonstrated statistically significant associations (*P* < 0.05), indicating their potential importance in the diagnostic process. The subsequent multivariate forward stepwise logistic regression affirmed age, size, aspect ratio, margin characteristics, microcalcifications, and duct dilatation as independent risk factors for breast cancer.

Age is a critical factor influencing malignant diseases. The global distribution of breast cancer incidence in women shows that breast cancer incidence is predominantly concentrated in individuals aged 45 and above, accounting for over 80% of all patients (19–21). Among individuals aged 25 and older, there is a rising trend in overall incidence rates with increasing age. The disparity in age between IDP and DCIS may be attributed to the varying peak ages of affliction for these two diseases. Regional data on the prime age for IDP incidence vary, with occurrences being more common across all age groups of women. A retrospective analysis of 4450 IDP patients revealed an average age of onset at 47.86 ± 11.93 years (22). Our study included 210 patients with IDP grades 3, 4, and 5, averaging at 44.81 ± 8.86 years, aligning closely with other research findings. Conversely, the median age for DCIS is 55 years, typically lower by 3-5 years compared to invasive breast cancer (23). In women under 30 years of age, DCIS is exceedingly uncommon, with its incidence increasing with advancing age (24). Among the 179 patients with mass-type DCIS included in our study, the average age was 48.2 ± 9.35 years, relatively lower than reported in other studies, possibly indicating increased participation in widespread screening programs in recent years. In our study, IDP had an average diameter of 1.26 ± 0.77 cm, often manifesting as small breast lesions with limited growth evident on ultrasound, possibly linked to its growth rate, tissue structure, and cell proliferation activity. In contrast, DCIS displayed notably larger diameters, suggestive of a more extensive spread and increased cellular atypia within breast tissue. Research indicates that patients with larger tumors (>20 mm), higher histological grades, and calcifications detected on mammography are more likely to experience pathological upgrading. Conversely, patients with smaller masses (<20 mm), well-differentiated tissue, and no calcifications on mammography exhibit lower risks of pathological upgrading (25).

DCIS exhibits a higher aspect ratio (>1) than IDP, with more indistinct margins and increased microcalcifications. Due to its invasiveness, DCIS shows non-uniform growth in various directions, whether within smaller matrices or infiltrating adjacent tissues, leading to an increased aspect ratio and a visually higher aspect ratio in imaging. Up to a quarter of DCIS lesions are diagnosed as invasive cancer upon core needle biopsy (26). The direct invasion of breast ductal tissue by IDP, along with its induction of reactive changes in surrounding tissues such as inflammatory responses and fibrosis, results in unclear boundaries between the tumor and neighboring tissues, causing blurred margins in imaging. In contrast, DCIS is confined within breast ducts, typically presenting as rounded or elliptical shapes, demonstrating slower growth and clearer boundaries. Additionally, calcifications are typical features of breast DCIS. A study involving the mammographic examination of 2141 DCIS cases revealed the presence of microcalcifications in 87% of the lesions (27). In our investigation, 60.3% of DCIS cases exhibited microcalcifications. This disparity could potentially stem from our exclusion of non-mass-like DCIS lesions as well as the lower sensitivity of ultrasound in detecting microcalcifications compared to mammography. A statistically significant variance was observed in the comparison of calcifications between the two cohorts. Calcifications are linked to the mineralization of necrotic cells and epithelial cell necrosis within tumors. DCIS lesions in the breast exhibit accelerated cellular proliferation rates relative to benign conditions, rendering them more susceptible to necrosis and subsequent calcification deposition.

A nomogram is the most common representation in clinical prediction models (28), excelling in incorporating multiple risk factors and transforming complex regression equations into graphical forms, ultimately enhancing the model’s predictive accuracy. In this study, a nomogram was constructed based on the aforementioned factors to differentiate between DCIS and IDP in the breast. The nomogram model demonstrated excellent discriminative power on the ROC curve, with the calibration curve indicating strong alignment between the model’s predicted probabilities for identifying breast DCIS and the actual occurrence rates. Additionally, both the calibration curve and clinical decision curve confirmed the model’s consistency and clinical utility. In clinical practice, histopathological results following biopsy serve as the gold standard for a patient’s definitive diagnosis. This nomogram can potentially reduce the utilization of invasive procedures such as preoperative biopsies, providing guidance on the selection of clinical surgical approaches.

Despite the advances made in this study, several limitations warrant consideration. The retrospective nature of the cohort study may introduce inherent biases and limit the generalizability of the results. The sample size, though substantial, may benefit from expansion to enhance statistical power and validate the nomogram across diverse populations. Additionally, biopsy is considered the gold standard for diagnosis, it has certain limitations, including the potential for sampling error, interpretation variability among pathologists, and the invasiveness of the procedure. Future research should explore its applicability across different subsets of patients with DCIS, particularly those with non-mass DCIS or larger lesions. We emphasize the need for prospective studies and multi-center trials to evaluate the robustness and applicability of the model across diverse populations and clinical environments. Conducting large-scale, multi-center clinical trials will provide strong evidence for the reliability of the nomogram in practical applications.

While our study successfully developed a nomogram for predicting outcomes in DCIS with statistical significance, it is essential to validate its real-world performance. Implementing AI-driven models in clinical practice poses challenges that extend beyond the development of algorithms. These challenges include ensuring seamless integration within existing clinical workflows, validating the model across diverse clinical environments, and promoting its widespread adoption among practitioners. Future research endeavors could address these limitations by conducting prospective studies to corroborate the nomogram’s predictive performance in real-time clinical settings. Incorporating additional imaging modalities, such as mammography and magnetic resonance imaging (MRI) (29, 30), along with molecular markers (31, 32), could offer a more comprehensive diagnostic approach. Exploring how these modalities complement the ultrasound features may enhance diagnostic accuracy and improve patient management. Continued efforts in refining predictive models and incorporating multi-modal approaches hold promise for advancing personalized care in breast cancer diagnosis and management. Collaboration between radiologists, pathologists, and oncologists to further optimize diagnostic algorithms and enhance patient outcomes remains paramount in the ongoing pursuit of precision medicine in breast cancer research.

## Conclusion

In conclusion, our study highlights essential ultrasound features that distinguish IDP from DCIS. Age, lesion size, aspect ratio, margin characteristics, microcalcifications, and duct dilatation emerged as independent predictors for the development of breast cancer. These findings pave the way for the construction of a predictive nomogram, which could enhance diagnostic accuracy and guide clinical management in breast cancer detection and treatment. The integration of these variables into routine ultrasound evaluations has the potential to significantly improve the early detection and differentiation of breast pathologies, ultimately contributing to better patient outcomes. Incorporating this tool into clinical practice may not only enhance decision-making and patient management but also potentially lead to changes in workflow and improvements in cost-effectiveness. Future research should focus on conducting prospective studies and multi-center trials to validate the nomogram’s effectiveness across diverse populations and clinical settings.

## Data Availability

The raw data supporting the conclusions of this article will be made available by the authors, without undue reservation.
