# Supplementary material for: Ultrasound characteristics comparison and development of a predictive nomogram for intraductal papilloma and ductal carcinoma in situ: a retrospective cohort study
Source: Front Oncol. 2025 Apr 17;15:1454951. doi: 10.3389/fonc.2025.1454951 (PMC12043480; doi:10.3389/fonc.2025.1454951)
Supplement: Supplementary file 1 [file DataSheet1.doc]

getwd()

setwd("C:/Users/10136/Desktop/R work/DCIS")

library(readr)

mydata <- read_csv("RT.csv")

View(mydata)

names(mydata)

mydata<-na.omit(mydata)

str(mydata)

attach(mydata)

mydata[,c(6:7,9:18)] <- lapply(mydata[,c(6:7,9:18)], factor)

library(compareGroups)

table1<-descrTable(cancer ~ ., data = mydata,show.all = TRUE)

print(table1)

export2xls(table1,file = "table1.xls")

dev = mydata[mydata$dataset==1,]

vad = mydata[mydata$dataset==0,]

table11<-descrTable(cancer~ .-dataset, data = mydata,subset = dataset == 1,show.all = TRUE)

print(table11)

export2csv(table11,file = "table6.csv")

table7<-descrTable(cancer ~ .-dataset, data = mydata,subset = dataset == 0,show.all = TRUE)

print(table7)

export2csv(table7,file = "table7.csv")

uni_glm_model<-function(x){

FML<-as.formula(paste0("cancer==1~",x))

glm1<-glm(FML,data = dev,family = binomial)

glm2<-summary(glm1)

OR<-round(exp(coef(glm1)),2)

SE<-round(glm2$coefficients[,2],3)

CI2.5<-round(exp(coef(glm1)-1.96*SE),2)

CI97.5<-round(exp(coef(glm1)+1.96*SE),2)

CI<-paste0(CI2.5,'-',CI97.5)

B<-round(glm2$coefficients[,1],3)

Z<-round(glm2$coefficients[,3],3)

P<-round(glm2$coefficients[,4],3)

uni_glm_model<-data.frame('characteristics'=x,

'B'=B,

'SE'=SE,

'OR'=OR,

'CI'=CI,

'Z' =Z,

'P'=P)[-1,]

uni_glm_model$characteristics=rownames(uni_glm_model)

return(uni_glm_model)

}

variable.names<-colnames(dev)[c(3:18)]

variable.names

uni_glm<-lapply(variable.names,uni_glm_model)

uni_glm

library(plyr)

library(dplyr)

uni_glm<-ldply(uni_glm,data.frame)

uni_glm

View(uni_glm)

write.csv(uni_glm, "uni.csv")

fml<-

as.formula(paste0('cancer==1~',paste0(uni_glm$characteristics[uni_glm$P<0.1],collapse = '+')))

fml

#modelB<-step(modelA,direction="forward")

#summary(modelB)

modelX<-glm(cancer==1~1,data = dev,family=binomial)

modelX

modelB<-step(modelX,scope=list(upper=~ age + dia + form + direction + boundary + surrounding + cal + duct + pblood,

lowe=~1), data = dev,family=binomial,direction ="forward")

summary(modelB)

fml <- as.formula(paste0('cancer == 1 ~ ', paste(c("age", "dia", "form", "direction", "boundary", "surrounding", "cal", "duct", "pblood"), collapse = ' + ')))

modelA<-glm(fml,data = dev,family=binomial)

modelB<-step(modelA,direction = "both")

modelB

glm3<-summary(modelB)

glm3

glm3$coefficients

OR<-round(exp(glm3$coefficients[,1]),2)

OR

SE<-round(glm3$coefficients[,2],3)

CI2.5<-round(exp(coef(modelB)-1.96*SE),2)

CI97.5<-round(exp(coef(modelB)+1.96*SE),2)

CI<-paste0(CI2.5,'-',CI97.5)

#CI

B<-round(glm3$coefficients[,1],3)

Z<-round(glm3$coefficients[,3],3)

P<-round(glm3$coefficients[,4],3)

mlogit<-data.frame(

'B'=B,

'SE'=SE,

'OR'=OR,

'CI'=CI,

'Z' =Z,

'P'=P)[-1,]

mlogit

characteristics=rownames(mlogit)

characteristics

mlogit<-cbind(characteristics,mlogit)

mlogit

View(mlogit)

write.csv(mlogit, "multi.csv")

final<-merge.data.frame(uni_glm,mlogit,by='characteristics',all = TRUE,sort = T)

final

View(final)

write.csv(final, "final.csv")

fml8<-as.formula(cancer == 1 ~ age + dia + form + direction + boundary + surrounding +

cal + duct + pblood)

model8<-glm(fml8,data = dev,family = binomial(logit))

dev$predmodel8<- predict(newdata=dev,model8,"response")

vad$predmodel8<- predict(newdata=vad,model8,"response")

View(vad)

#install.packages("pROC")

library(pROC)

devmodelA <- roc(cancer~predmodel8, data = dev,smooth=F)

#devmodelA <- roc(hypoglycemia~predmodel8, data = dev,smooth=T)

devmodelA

round(auc(devmodelA),3)

round(ci(auc(devmodelA)),3)

plot(devmodelA,

print.auc=TRUE,

print.thres=TRUE,

legacy.axes=TRUE,

main = "devmodel",

col= "red",

print.thres.col="red",

identity.col="blue",

identity.lty=1,

identity.lwd=1)

vadmodelA <- roc(cancer~predmodel8, data = vad,smooth=F)

round(auc(vadmodelA),3)

round(ci(auc(vadmodelA)),3)

#ROC

plot(vadmodelA,

print.auc=TRUE,

print.thres=TRUE,

ain = "ROC CURVE",

col= "red",

print.thres.col="red",

identity.col="blue",

identity.lty=1,

identity.lwd=1)

library(calibrate)

library(MASS)

install.packages("rms")

library(rms)

fml<-cancer==1 ~age + dia + form + direction + boundary + surrounding +

cal + duct + pblood

fit3<-lrm(fml,data=dev,x=TRUE,y=TRUE)

cal3<-calibrate(fit3,method="boot",B=1000) #Bootstrap

plot(cal3,

xlim = c(0,1),

xlab = "Predicted Probability",

ylab = "Observed Probability",

legend=FALSE,

subtitles = FALSE)

abline(0,1,col="black",lty=2,lwd=2)

lines(cal3[,c("predy","calibrated.orig")],type = "l",lwd=2,col="red",pch=16)

lines(cal3[,c("predy","calibrated.corrected")],type = "l",lwd=2,col="green",pch=16)

legend(0.65,0.40,

c("Ideal","Apparent","Bias-corrected"),

lty = c(2,1,1),

lwd = c(2,1,1),

col = c("black","red","green"),

bty = "n")

fit4<-lrm(fit3,data=vad,x=TRUE,y=TRUE)

cal4<-calibrate(fit4,method="boot",B=1000)

plot(cal4,

xlim = c(0,1),

xlab = "Predicted Probability",

ylab = "Observed Probability",

legend=FALSE,

subtitles = FALSE)

abline(0,1,col="black",lty=2,lwd=2)

lines(cal4[,c("predy","calibrated.orig")],type = "l",lwd=2,col="red",pch=16)

lines(cal4[,c("predy","calibrated.corrected")],type = "l",lwd=2,col="green",pch=16)

legend(0.65,0.30,

c("Ideal","Apparent","Bias-corrected"),

lty = c(2,1,1),

lwd = c(2,1,1),

col = c("black","red","green"),

bty = "n")

getwd()

setwd("C:/Users/10136/Desktop/R work/DCIS")

library(readr)

mydata <- read_csv("RT.csv")

mydata<-na.omit(mydata)

attach(mydata)

dev = mydata[mydata$dataset==1,]

vad = mydata[mydata$dataset==0,]

model_1<-decision_curve(cancer ~ age + dia + form + direction + boundary + surrounding +

cal + duct + pblood,

data = dev,

family = binomial(logit),

thresholds = seq(0,1,by=0.01),

confidence.intervals = 0.95,

study.design = 'case-control',

population.prevalence =0.1)

plot_decision_curve(model_1,curve.names = c('Nomogram'),

xlim = c(0,1),

cost.benefit.axis = T,

col = c('red'),

confidence.intervals = F,

standardize = T)

fml8<-as.formula(cancer == 1 ~age + dia + form + direction + boundary + surrounding +

cal + duct + pblood)

model8<-glm(fml8,data = dev,family = binomial(logit))

dev$predmodel8<- predict(newdata=dev,model8,"response")

vad$predmodel8<- predict(newdata=vad,model8,"response")

vadmodel8 <- decision_curve(cancer~predmodel8,

data = vad,

fitted.risk = TRUE,

thresholds = seq(0, .9, by = .05),

bootstraps = 500)

plot_decision_curve(vadmodel8,curve.names = c('Nomogram'),

legend.position = "topright",

confidence.intervals = F, #remove confidence intervals)

standardize = T)

mydata$Aspect_ratio<-factor(mydata$Aspect_ratio,

levels = c(0,1),

labels=c("Aspect_ratio<1","Aspect_ratio≥1"))

mydata$Margin <-factor(mydata$Margin,

levels = c(0,1),

labels=c("distinct","indistinct"))

mydata$ Microcalcification<-factor(mydata$Microcalcification,

levels = c(0,1),

labels=c("no","yes"))

mydata$ Duct_dilatation<-factor(mydata$Duct_dilatation,

levels = c(0,1),

labels=c("no","yes"))

dev = mydata[mydata$dataset==1,]

vad = mydata[mydata$dataset==0,]

library(Hmisc)

library(rms)

ddist <- datadist(dev)

options(datadist='ddist')

#install.packages("regplot")

library(regplot)

c <- glm(cancer ~age + Size + Aspect_ratio + Margin + Microcalcification + Duct_dilatation, data = dev, family = binomial(link="logit"))

regplot(c,observation=dev[100,])

dev$Aspect_ratio<-factor(dev$Aspect_ratio,labels=c("Aspect_ratio<1","Aspect_ratio≥1"))

dev$Margin<-factor(dev$Margin,labels=c("distinct","indistinct"))

dev$Microcalcification<-factor(dev$Microcalcification,labels=c('No','Yes'))

dev$Duct_dilatation<-factor(dev$Duct_dilatation,labels=c('No','Yes'))

modelD<-glm(cancer ~ age + Size + Aspect_ratio + Margin + Microcalcification + Duct_dilatation,family = binomial,data=dev)

DynNom(modelD,DNtitle="Nomogram",DNxlab="probability",data = dev)

#动态发布

DNbuilder(modelD)

rsconnect::setAccountInfo(name='suliyang',

token='2F2F4AD1FDF1F61C6982828DE9C7F6AA',

secret='jw0GRsbDjZH0mJEYHlysoKeNRiawMcVaNF4AnRxT')

#https://songge.shinyapps.io/ICU_death/

fml8<-as.formula(cancer == 1 ~ symptom + size + margin + microcalcification + posterior_echo + halo + internal_flow )

model8<-glm(fml8,data = dev,family = binomial(logit))

dev$predmodel8<- predict(newdata=dev,model8,"response")

vad$predmodel8<- predict(newdata=vad,model8,"response")

#install.packages("calibrate")

library(calibrate)

library(MASS)

#install.packages("rms")

library(rms)

val.prob(dev$predmodel8,dev$cancer)

val.prob(vad$predmodel8,vad$cancer)

source("HLtest.R")

hl.ext2(dev$predmodel8,dev$hypoglycemia)

source("HLtest.R")

hl.ext2(vad$predmodel8,vad$cancer)

fml<-cancer==1 ~symptom + size + margin + microcalcification + posterior_echo + halo + internal_flow

fit3<-lrm(fml,data=dev,x=TRUE,y=TRUE)

cal3<-calibrate(fit3,method="boot",B=1000) #Bootstrap

plot(cal3,

xlim = c(0,1),

xlab = "Predicted Probability",

ylab = "Observed Probability",

legend=FALSE,

subtitles = FALSE)

abline(0,1,col="black",lty=2,lwd=2)

lines(cal3[,c("predy","calibrated.orig")],type = "l",lwd=2,col="red",pch=16)

lines(cal3[,c("predy","calibrated.corrected")],type = "l",lwd=2,col="green",pch=16)

legend(0.55,0.35,

c("Ideal","Apparent","Bias-corrected"),

lty = c(2,1,1),

lwd = c(2,1,1),

col = c("black","red","green"),

bty = "n")

source("HLtest.R")

hl.ext2(dev$fit3,dev$cancer)
